# Parliament and the Doctors

**Published:** 1917-06-30

**Authors:** 


### THE ATTACKS ON MEDICAL RECRUITING OFFICERS.

### Parliament and the Doctors.

The war has set in a fierce light many institutions, methods, and individuals, and among these must certainly be counted the medical profession, both in its corporate capacity and in the person of some of its members. In its most conspicuous relation to the national emergency, that is in the supply of medical officers for the fighting forces, the profession has emerged with added credit from the test, for, admittedly, large sacrifices have been made, effective service has been rendered, and the battlefield itself has sealed the record. The good health of our armies and the care of our sick and wounded certainly speak in no uncertain tones of the skill, knowledge, enterprise, and adaptability which have marked the work of the civilian practitioners who now form the enormous majority of the R.A.M.C. Moreover, recognition of all this has not been wanting either from the official world or from public opinion, and if the praise has not always been according to knowledge, it has on the whole been well merited. The profession, however, is saved from the woe promised for those of whom all men speak well, and if any doubt remained on this point it was dissipated by the recent debate in the House of Commons on the medical examination of recruits. Of the charges made in the course of this debate it is necessary to take serious note, for if they are well founded, they must bring, and deservedly, heavy censure on the practitioners concerned.

The charges fall into two classes. The first of these includes an accusation of professional incompetence, or at least of professional carelessness. The second alleges harsh or even brutal treatment of the men who present themselves for examination. We make no complaint that such statements should be ventilated in the House of Commons. On the contrary, we agree that it is in accordance with justice that they should be published openly and should be brought to the test of investigation. Further, we allow, and without any shadow of reservation, that the members of the House who made themselves responsible for the indictment were animated by a sense of public duty, and by a sincere desire to protect individuals from suffering and wrong. Possibly minds of a generous tone would have more freely recognised that the instances quoted were exceptional, and that behind them stood a large body of efficient and difficult work discharged in a courteous, considerate, and patriotic fashion. But this is a matter of taste on which no quarrel need be sought. The essential thing is to discover whether these charges are true, and if true, to prevent future occurrences of the same order. We entirely agree that the question cannot be closed by an official quotation of the smooth generalities of inspectors appointed to travel about the country and to visit, in the newly acquired dignity of military state, the recruiting boards and stations. The charges are specific and particular, and can only be met by evidence which is strictly relevant to the individual issues. Hence we welcome the appointment of a Parliamentary Committee, and we trust that here both accusers and accused will meet on equal terms, and will be summoned to discharge their respective responsibilities. The profession generally, we are convinced, desires only that the truth shall be discovered, and has not the slightest desire to shelter or protect any of its members who shall be proved guilty either of incompetence, or of carelessness, or of discourtesy. For our own part, we are satisfied, without expressing any opinion on the instances now in question, that the great majority of the medical officers responsible for the examination of recruits have discharged their duties with efficiency and goodwill, and have, at the cost of no slight sacrifice, deserved well of their country.

There are one or two considerations relative to the general question which may perhaps usefully be repeated. First, in the very nature of things, as the work is performed by fallible instruments, a certain proportion of mistakes is inevitable. Again, in certain of the problems presented there is room for honest differences of opinion between men of equal good faith and equal competence, and in this way are explained at least some of the cases in which different boards have, at different times, placed one and the same man in different orders of classification. To read into such events invincible ignorance, carelessness, or stupidity is merely to display a want of appreciation of the essential difficulties of the position. Further, it is not improbable that pressure of time has occasionally clashed with thoroughness of examination, and though such an excuse is devoid of validity, it is difficult completely to eliminate it from the world of practical affairs; nor is the responsibility for such a state of matters entirely with the medical officers. Again, it must be remembered that these officers have a duty to the country as well as to the individuals who come under their review. That all of these individuals should receive care and courtesy goes without saying, and there is indeed a special appeal in the case of men who have already served to their hurt in the fighting ranks and who are called up for re-examination. On the other hand, the national cause must have men to uphold it if it is not to come to disaster, and the duty of equal balance must be discharged however pathetic may be the individual claim. The public must recollect also that the need is not confined to actual fighters, and that much help of a less strenuous order is essential if the Army is to continue in being; and again, that the private judgment of his capacity by the person immediately concerned is neither an impartial nor a competent one. By those who know the facts the duties of the medical officer engaged in the examination of recruits are recognised as by no means devoid of difficulty, and while default, we quite agree, ought to meet with censure, honest and valuable service in this direction deserves a somewhat more cordial recognition than it has hitherto received.

